# Interferon-γ-dependent control of *Anaplasma phagocytophilum* by murine neutrophil granulocytes

**DOI:** 10.1186/s13071-017-2274-6

**Published:** 2017-07-12

**Authors:** Kathrin Gussmann, Susanne Kirschnek, Friederike D. von Loewenich

**Affiliations:** 1 Institute of Medical Microbiology and Hygiene, University of Freiburg, Hermann-Herder-Strasse 11, D-79104 Freiburg, Germany; 2 Department of Medical Microbiology and Hygiene, University of Mainz, Obere Zahlbacherstrasse 67, D-55131 Mainz, Germany

**Keywords:** *Anaplasma phagocytophilum*, Hoxb8, Inducible nitric oxide synthase, Interferon-γ, Myeloperoxidase, NADPH-oxidase, Neutrophil

## Abstract

**Background:**

*Anaplasma phagocytophilum* is a Gram-negative obligate intracellular bacterium that is transmitted by ticks of the *Ixodes ricinus* complex. It replicates in neutrophils and elicits febrile disease in humans and animals. Because of its striking tropism for neutrophils, *A. phagocytophilum* has been used as a model organism to study the immune response against obligate intracellular pathogens. In mice, the control of *A. phagocytophilum* in the early phase of infection is dependent on natural killer cell-derived interferon-γ (IFN-γ). In contrast, the final elimination strictly requires CD4^+^ T-cells. It is a matter of debate, whether neutrophils serve only as host cells or as killer cells as well.

**Results:**

To study this, we used in vitro generated murine neutrophils with defects in major antimicrobial molecules such as NADPH-oxidase (gp91^phox−/−^), myeloperoxidase (MPO^−/−^) and inducible nitric oxide synthase (iNOS^−/−^). However, bacterial growth in gene-deficient neutrophils was comparable to that in wild-type cells. Whereas gp91^phox^ and MPO expression remained unchanged, the infection led to an induction of iNOS. In neutrophils stimulated with IFN-γ, bacterial growth was significantly impaired, and iNOS was induced. However, the antibacterial effect of IFN-γ was still seen in iNOS^−/−^ neutrophils.

**Conclusion:**

Thus, murine in vitro generated neutrophils stimulated with IFN-γ seem to act as killer cells by an iNOS-independent mechanism.

**Electronic supplementary material:**

The online version of this article (doi:10.1186/s13071-017-2274-6) contains supplementary material, which is available to authorized users.

## Background

*Anaplasma phagocytophilum* is a Gram-negative obligate intracellular bacterium [1] that is transmitted by ticks of the *Ixodes ricinus* complex [2]. In contrast to the assumption of previous reports, the direct human-to-human transmission does not occur [3]. It replicates primarily in neutrophils [4] and elicits febrile disease in humans [5], domestic ruminants [6], dogs [7], horses [8] and cats [9]. In humans, the most prevalent symptoms comprise fever, headache, myalgias and arthralgias [5]. The lethality is 0.6% [10].

Because of its striking tropism for neutrophils, *A. phagocytophilum* has been used as a model organism to study the immune response against obligate intracellular pathogens. Using gene-deficient mice, it became clear that interferon-γ (IFN-γ) is important in the early control of *A. phagocytophilum* but dispensable for final elimination [11–14]. We showed that in the early phase of infection natural killer (NK) cells are the main source of IFN-γ that is probably induced by type I interferon and interleukin (IL)-12 [12]. However, others reported that NKT cells [15] and IL12/IL18 activated CD4^+^ T cells contribute to the early IFN-γ production as well [16, 17]. In line with the finding in mice, humans with granulocytic anaplasmosis show elevated IFN-γ levels in their acute-phase sera [18]. Although the final clearance of *A. phagocytophilum* strictly depends on CD4^+^ T-cells, the underlying mechanism is unclear to date [12].

Whether neutrophils serve only as host cells or contribute to the killing of the pathogen, is still a matter of debate [4]. In vivo, major antimicrobial molecules of neutrophils such as NADPH-oxidase, myeloperoxidase (MPO), inducible nitric oxide synthase (iNOS), granulocyte elastase and cathepsin G were dispensable for the control of *A. phagocytophilum* [12, 19]. In vitro, reactive oxygen species (ROS), which are produced by the phagocyte NADPH-oxidase [20], were not induced in primary human neutrophils stimulated with *A. phagocytophilum* [21–24]. Whether *A. phagocytophilum* actively suppresses ROS production in primary human neutrophils is a matter of debate [21, 23, 24]. However, it has been shown that it scavenges O_2_^−^ thereby protecting itself [23, 24].

In vivo, the replication of *A. phagocytophilum* strictly depends on neutrophils [12] though their major antimicrobial molecules are dispensable for pathogen elimination [12, 19]. However, because of the redundancy of the immune system, in vivo, the defect in one defence mechanism might be compensated by the other. Therefore, we infected in vitro generated murine neutrophils with defects in NADPH-oxidase, MPO and iNOS with *A. phagocytophilum* and compared the course of infection to it in wild-type cells. To do so, murine neutrophil progenitor cells were immortalised by the estrogen-regulated Hoxb8 oncogene [25]. After estrogen-withdrawal, the progenitor cells differentiate into mature neutrophils that are almost indistinguishable from primary murine neutrophils [25–27].

We show here that NADPH-oxidase, MPO and iNOS do not contribute to the control of *A. phagocytophilum* in vitro. However, IFN-γ had an antibacterial effect on *A. phagocytophilum* replicating in Hoxb8 neutrophils.

## Results

### Growth of *A. phagocytophilum* in Hoxb8 neutrophils

**Fig. 1:**
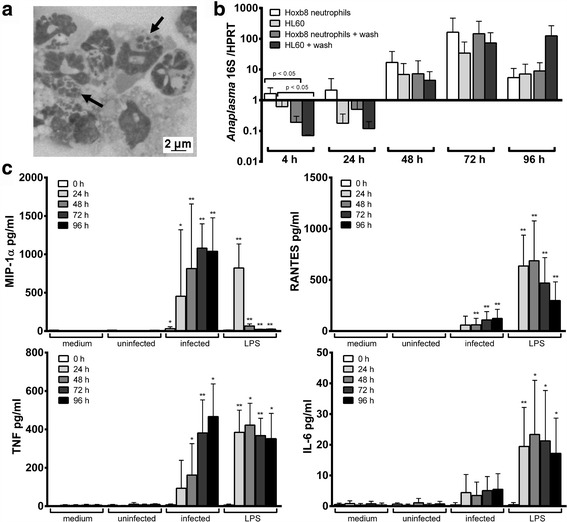
**a** Diff-Quick stain of *A. phagocytophilum* Webster strain in Hoxb8 neutrophils. Bacteria were grown 3 days in Hoxb8 neutrophils, cytocentrifuged onto a glass slide and stained by Diff-Quick (magnification ×1000; *scale-bar*: 2 μm). Arrows: morulae of *A. phagocytophilum* inside Hoxb8 neutrophils. **b** Increase of *A. phagocytophilum* 16S rRNA relative to murine HPRT mRNA at different time points after infection of Hoxb8 neutrophils. Results were normalised to the respective 0 h value of each sample using the ΔΔC_t_-method. The inoculum was prepared from infected Hoxb8 neutrophils or infected HL60 cells. Half of the set of samples was washed 2× in PBS at 4 h p.i. and subsequently supplied with fresh medium. Mean and SD from 4 independent experiments are shown. Differences between experimental groups were analysed using the two-tailed Mann-Whitney test. The following groups were compared: Hoxb8 neutrophils *vs *HL60 cells (non-significant), Hoxb8 neutrophils + wash *vs* HL60 cells + wash (non-significant), Hoxb8 neutrophils *vs* Hoxb8 neutrophils + wash (significant only at 4 h p.i., *P* < 0.05) and HL60 cells *vs* HL60 cells + wash (significant only at 4 h p.i., *P* < 0.05). **c** Chemokine and cytokine production in unstimulated (medium) Hoxb8 neutrophils, in Hoxb8 neutrophils stimulated with uninfected lysed Hoxb8 neutrophils (uninfected), in Hoxb8 neutrophils stimulated with *A. phagocytophilum*-infected lysed Hoxb8 neutrophils (infected) or in Hoxb8 neutrophils stimulated with 10 ng/l LPS at different time points. MIP-1α, RANTES, TNF and IL-6 were measured in the supernatants using CBA assay. Mean and SD from 5 independent experiments are shown. Differences between experimental groups were analysed using the two-tailed Mann-Whitney test. Hox8 neutrophils treated with lysed uninfected and lysed infected Hoxb8 neutrophils or LPS were compared to those treated with medium only. *P < 0.05, **P < 0.01

The human promyelocytic leukaemia cell line HL60 is routinely used to propagate *A. phagocytophilum* [28]. Therefore, first of all, *A. phagocytophilum* Webster strain grown in HL60 cells was used to infect murine Hoxb8 neutrophils. It grew without difficulty (Fig. 1a), what supports previous findings that Hoxb8 neutrophils functionally resemble primary murine neutrophils [25–27]. Next, we tested whether the growth characteristics were dependent on the origin of the inoculum. For this purpose, we infected Hoxb8 neutrophils with *A. phagocytophilum* cultured in murine Hoxb8 neutrophils or human HL60 cells. As shown in Fig. 1b there were no significant differences relying on the source of the bacteria. Therefore, for further experiments, inocula were prepared from infected Hoxb8 neutrophils.

The invasion of *A. phagocytophilum* in human neutrophils has been shown to take up 4 to 6 h [23, 24, 29]. Therefore, two additional sets of samples were washed after 4 h to remove un-invasive bacteria. Significant differences were only found between washed and unwashed samples at 4 h post-infection (p.i.) (*U* = 0.0, *n*_1_ = *n*_2_ = 4, *P* = 0.0286 (Hoxb8 neutrophils), *P* = 0.0294 (HL60 cells), Fig. 1b). Thus, further experiments were performed without the washing step.

### Chemokine and cytokine production by wild-type Hoxb8 neutrophils

Depending on the stimulus, murine and human neutrophils can produce significant amounts of chemokines and cytokines [30]. Therefore, IFN-γ, IL-1β, IL-6, IL-10, IL-12/IL-23p40, IL-17A, KC (CXCL1), MCP-1 (CCL2), MIG (CXCL9), MIP-1α (CCL3), RANTES (CCL5) and TNF were measured in the supernatants of *A. phagocytophilum-*infected or LPS-stimulated wild-type Hoxb8 neutrophils. Whereas IFN-γ, IL-1β, IL-10, IL-12/IL-23p40, IL-17A, KC and MIG were not produced after infection or LPS-stimulation, elevated MCP-1 levels were measured only at 72–96 h (data not shown). In contrast, statistically significant higher amounts of MIP-1α, RANTES and TNF compared to the medium control were found in the supernatants of *A. phagocytophilum*-infected and LPS-stimulated Hoxb8 neutrophils (Fig. 1c). IL-6 levels were elevated only after LPS-stimulation. Therefore, MIP-1α, RANTES, TNF and IL-6 were chosen for further analyses. In summary, the results show that an *A. phagocytophilum* infection leads to a stimulation of its host cells that the bacterium is not able to fully suppress.

### Impact of antimicrobial effector mechanisms of neutrophils on the growth of *A. phagocytophilum*

The control of *A. phagocytophilum* in vivo is independent of NADPH-oxidase, MPO and iNOS [12, 19]. However, in vivo, a defect could be compensated by the action of other immune cells or at the neutrophil level by the compensatory up-regulation of other effector mechanisms. Therefore, the growth of *A. phagocytophilum* in Hoxb8 neutrophils defective for NADPH-oxidase (gp91^phox^), MPO and iNOS was compared to it in wild-type cells. Further, expression of the respective mRNAs and nitrite production were measured after infection or LPS-stimulation using LPS as a positive control.

**Fig. 2:**
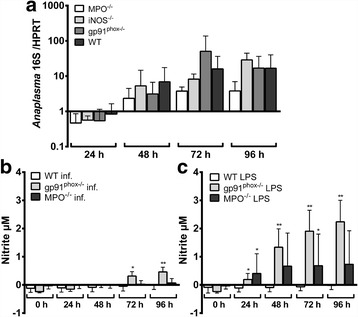
**a** Increase of *A. phagocytophilum* 16S rRNA relative to murine HPRT mRNA at different time points p.i. of MPO^−/−^, iNOS^−/−^, gp91phox^−/−^ and wild-type (WT) neutrophils. Results were normalised to the respective 0 h value of each sample using the ΔΔC_t_-method. Mean and SD from 7 independent experiments are shown. Differences between experimental groups were analysed using the two-tailed Mann-Whitney test. The following groups were compared: WT and MPO^−/−^, iNOS^−/−^, gp91phox^−/−^ neutrophils, respectively at each time point. Statistically significant differences were not detected. **b**, **c** Nitrite accumulation as a marker for iNOS activity in the supernatants of *A. phagocytophilum*-infected (**b**) or LPS-stimulated (10 ng/ml) (**c**) WT, gp91^phox−/−^ and MPO^−/−^ Hoxb8 neutrophils measured by Griess assay. Nitrite production could not be detected in all uninfected samples or in iNOS^−/−^ cells (data not shown). Mean and SD from 7 independent experiments are presented. Differences between experimental groups were analysed using the two-tailed Mann-Whitney test. Infected and LPS-stimulated WT Hoxb8 neutrophils were compared to the respective gp91^phox−/−^ and MPO^−/−^ cells at each time point. *P < 0.05, **P < 0.01

**Fig. 3:**
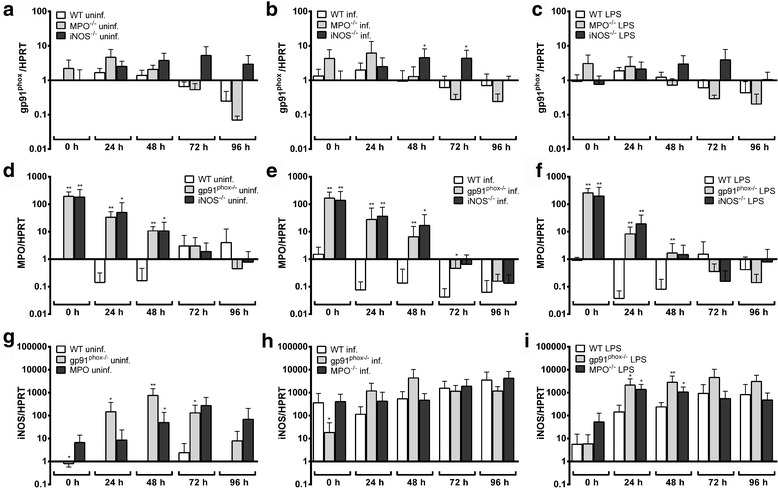
**a**-**c** Relative gp91^phox^ mRNA expression normalised to murine HPRT at different time points in uninfected (**a**), *A. phagocytophilum*-infected (**b**) and LPS-stimulated (10 ng/ml) (**c**) wild-type (WT), MPO^−/−^ and iNOS^−/−^ Hoxb8 neutrophils. Results were normalised to the 0 h value of uninfected WT cells using the ΔΔC_t_-method. Mean and SD from 7 independent experiments are shown. Differences between experimental groups were analysed using the two-tailed Mann-Whitney test. The following groups were compared: MPO^−/−^ and iNOS^−/−^ Hoxb8 neutrophils to WT cells at each time point. *P < 0.05. **d**-**f** Relative MPO mRNA expression normalised to murine HPRT at different time points in uninfected (**d**), *A. phagocytophilum*-infected (**e**) and LPS-stimulated (10 ng/ml) (**f**) WT, gp91^phox−/−^ and iNOS^−/−^ Hoxb8 neutrophils. Results were normalised to the 0 h value of uninfected WT cells using the ΔΔC_t_-method. Mean and SD from 7 independent experiments are shown. Differences between experimental groups were analysed using the two-tailed Mann-Whitney test. The following groups were compared: gp91^phox−/−^ and iNOS^−/−^ Hoxb8 neutrophils to WT cells at each time point. *P < 0.05, **P < 0.01. **g**-**i** Relative iNOS mRNA expression normalised to murine HPRT at different time points in uninfected (**g**), *A. phagocytophilum*-infected (**h**) and LPS-stimulated (10 ng/ml) (**i**) WT, gp91^phox−/−^ and MPO^−/−^ Hoxb8 neutrophils. Results were normalised to the 0 h value of uninfected WT cells using the ΔΔC_t_-method. Mean and SD from 7 independent experiments are shown. Differences between experimental groups were analysed using the two-tailed Mann-Whitney test. The following groups were compared: gp91^phox−/−^ and MPO^−/−^ Hoxb8 neutrophils to WT cells at each time point. *P < 0.05, **P < 0.01

As shown in Fig. 2a, there was no significant difference in the bacterial growth in Hoxb8 wild-type and knock-out neutrophils. gp91^phox^ mRNA was expressed in uninfected wild-type Hoxb8 neutrophils (μ = C_t_ 26.5, SD = C_t_ 2.1, 7 experiments). Neither infection nor LPS-stimulation led to a significant induction of gp91^phox^ mRNA in wild-type (Additional file 1: Figure S1a), MPO^−/−^ (Additional file 1: Figure S1b) or iNOS^−/−^ (Additional file 1: Figure S1c) Hoxb8 neutrophils. However, slightly (5- to 8-fold), but significantly elevated gp91^phox^ mRNA levels were found in iNOS^−/−^ cells at 48 and 72 h p.i. when compared to wild-type neutrophils (*U* = 3, *n*_1_ = 4, *n*_2_ = 7, *P* = 0.0424, Fig. 3b). There were no statistically significant differences in gp91^phox^ mRNA levels between uninfected (Fig. 3a) or LPS-stimulated (Fig. 3c) wild-type and MPO^−/−^ or iNOS^−/−^ cells.

MPO mRNA was only weakly expressed in uninfected wild-type Hoxb8 neutrophils (μ = C_t_ 30.1, SD = C_t_ 1.5, 7 experiments). This is in line with the fact that the MPO synthesis is initiated at the promyelocyte stage and terminates at the myelocyte stage of neutrophil development [31]. Further, MPO mRNA was not induced due to infection or LPS-stimulation in wild-type (Additional file 1: Figure S1d), gp91^phox−/−^ (Additional file 1: Figure S1e) or iNOS^−/−^ cells (Additional file 1: Figure S1f). However, in gp91^phox−/−^ and iNOS^−/−^ Hoxb8 neutrophils the MPO mRNA expression was 10- to 100-fold elevated when compared to wild-type cells at the time points 0–48 h, but the effect was equally present in uninfected (Fig. 3d), infected (Fig. 3e) and LPS-stimulated Hoxb8 neutrophils (Fig. 3f).

iNOS mRNA was hardly expressed in uninfected wild-type Hoxb8 neutrophils (μ = C_t_ 39.4, SD = C_t_ 1.7, 7 experiments), but was 100- to 1000-fold induced upon infection or LPS-stimulation at the time points 24–96 h (*U* = 0, *n*_1_ = *n*_2_ = 7, *P* = 0.0006 at 24 and 48 h p.i., Additional file 1: Figure S1g). This effect was not statistically significant in gp91^phox−/−^ (Additional file 1: Figure S1 h) or MPO^−/−^ (Additional file 1: Figure S1i) Hoxb8 neutrophils at most time points, because the basal iNOS mRNA expression was already significantly higher in uninfected gp91^phox−/−^ and MPO^−/−^ cells when compared to wild-type cells at least at some time points (Fig. 3g). Statistically significant higher iNOS mRNA levels in gp91^phox−/−^ and MPO^−/−^ Hoxb8 neutrophils compared to wild-type cells were not found upon infection (Fig. 3h), but at 24 h (*U* = 1, *n*_1_ = 4, *n*_2_ = 7, *P* = 0.0121) and 48 h (gp91^phox−/−^ cells: *U* = 0, *n*_1_ = 4, *n*_2_ = 7, *P* = 0.0061; MPO^−/−^ cells: *U* = 2, *n*_1_ = 4, *n*_2_ = 7, *P* = 0.0242) after LPS-stimulation (Fig. 3i).

Because iNOS mRNA in contrast to gp91^phox^ and MPO mRNA was strongly induced after infection or LPS-stimulation, nitrite accumulation as a marker for iNOS activity was measured in the supernatants. In all medium controls and in all samples from iNOS^−/−^ Hoxb8 neutrophils nitrite production was not detectable (data not shown). Significantly elevated amounts of nitrite were only present in *A. phagocytophilum*-infected gp91^phox−/−^ cells at 72–96 h and in LPS-stimulated gp91^phox−/−^ Hoxb8 neutrophils at 24–96 h when compared to the medium controls of the respective time points (*U* = 0, *n*_1_ = 4, *n*_2_ = 4, *P* = 0.0286, data not shown). Infected gp91^phox−/−^ cells produced significantly more nitrite than infected wild-type Hoxb8 neutrophils at 72 h (*U* = 1, *n*_1_ = 4, *n*_2_ = 7, *P* = 0.0121) and 96 h (*U* = 0, *n*_1_ = 4, *n*_2_ = 7, *P* = 0.0061) p.i. (Fig. 2b). The same was not true for MPO^−/−^ cells. However, when stimulated with LPS, significantly elevated nitrite levels were found in gp91^phox−/−^ and MPO^−/−^ Hoxb8 neutrophils at at least some time points (Fig. 2c).

In conclusion, the unaltered growth of *A. phagocytophilum* in gp91^phox−/−^, MPO^−/−^ and iNOS^−/−^ Hoxb8 neutrophils suggests that the pathogen is either insensitive to reactive oxygen or nitrogen species or that the neutrophil can compensate for the defect. However, gp91^phox^ mRNA expression was essentially unaltered in MPO^−/−^ and iNOS^−/−^ Hoxb8 neutrophils in general. Further, the elevated MPO and iNOS mRNA expression in gp91^phox−/−^ and iNOS^−/−^ cells and in gp91^phox−/−^ and MPO^−/−^ cells, respectively was already present in uninfected cells and was not further increased in infected cells. Nitrite production in gp91^phox−/−^ Hoxb8 neutrophils was significantly, but slightly elevated compared to wild-type cells. Thus, it seems that the effector mechanisms tested here are not significantly involved in compensating for the respective defect at least in the context of an *A. phagocytophilum* infection.

### IFN-γ-dependent control of *A. phagocytophilum*

**Fig. 4:**
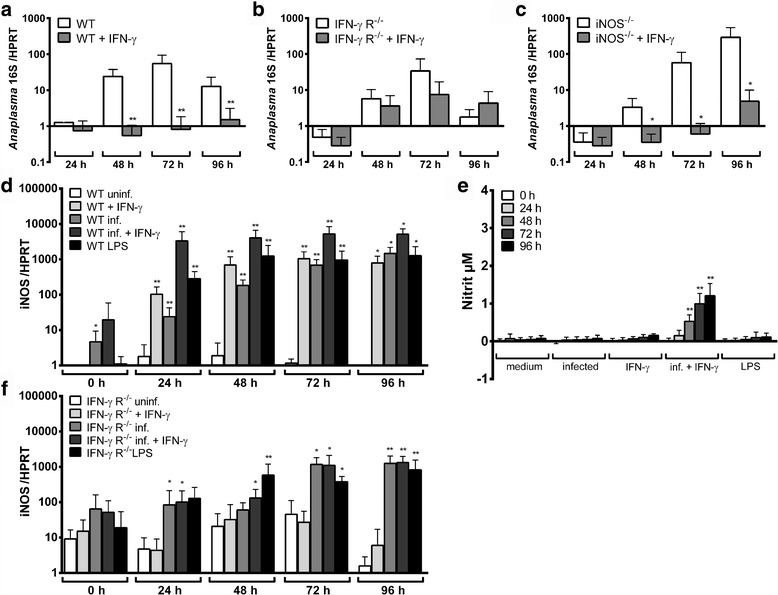
**a**-**c** Increase of *A. phagocytophilum* 16S rRNA relative to murine HPRT mRNA at different time points p.i. of wild-type (WT) (**a**), IFN-γ R^−/−^ (**b**) and iNOS^−/−^ (**c**) neutrophils. Half of the set of samples was stimulated with IFN-γ. Results were normalised to the respective 0 h value of each sample using the ΔΔC_t_-method. Mean and SD from 5 independent experiments are shown. Differences between unstimulated and IFN-γ-stimulated groups at each time point were analysed using the two-tailed Mann-Whitney test. *P < 0.05, **P < 0.01. **d**, **f** Relative iNOS mRNA expression normalised to murine HPRT at different time points in uninfected, IFN-γ-stimulated, *A. phagocytophilum*-infected, *A. phagocytophilum*-infected + INF-γ-stimulated, and LPS-stimulated (200 ng/ml) WT (**d**) and IFN-γ R^−/−^ (**f**) Hoxb8 neutrophils. Results were normalised to the 0 h value of uninfected WT cells using the ΔΔC_t_-method. Mean and SD from 5 independent experiments are shown. Differences between experimental groups were analysed using the two-tailed Mann-Whitney test. Stimulated and/or infected WT and IFN-γ R^−/−^ Hoxb8 neutrophils were compared to the respective uninfected controls. *P < 0.05, **P < 0.01. Further, a significant difference was detected between infected and infected + IFN-γ-stimulated WT cells at time points 24 h, 48 h, 72 h (*P* < 0.01) and 92 h (*P* < 0.05) p.i. The same was not true for IFN-γ R^−/−^ Hoxb8 neutrophils. **e** Nitrite accumulation as a marker for iNOS activity was measured in the supernatants of uninfected (medium), *A. phagocytophilum*-infected, IFN-γ-stimulated, *A. phagocytophilum*-infected + IFN-γ-stimulated, and LPS-stimulated (200 ng/ml) WT Hoxb8 neutrophils by Griess assay. Mean and SD from 5 independent experiments are shown. Differences between infected and/or stimulated cells and the medium controls at each time point were analysed using the two-tailed Mann-Whitney test. **P < 0.01

Next, as IFN-γ is known to activate neutrophil function [32] and to induce iNOS [33], we investigated whether INF-γ had a direct effect on the growth of *A. phagocytophilum* in Hoxb8 neutrophils. IFN-γ stimulation of wild-type cells led to a significantly reduced bacterial growth at 48–96 h p.i. when compared to unstimulated controls (*U* = 0, *n*_1_ = *n*_2_ = 5, *P* = 0.0079, Fig. 4a).

**Fig. 5:**
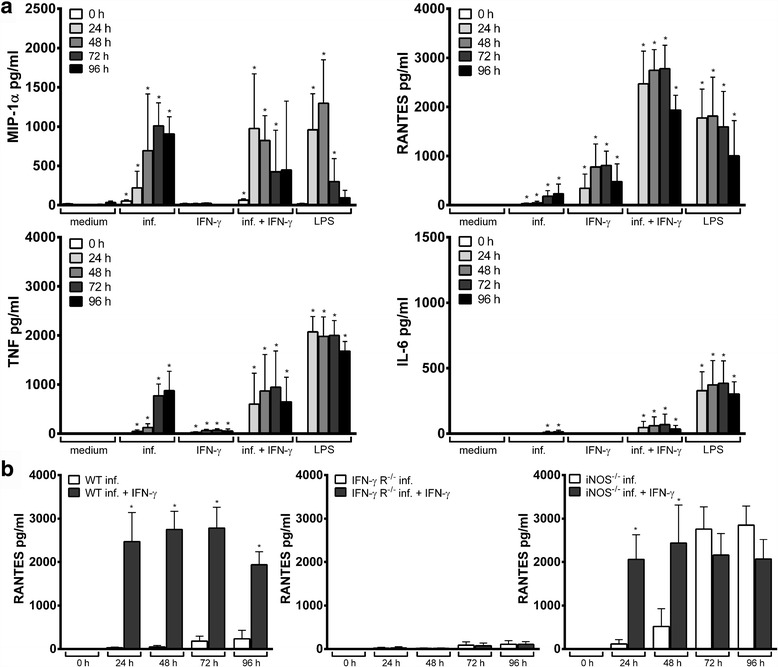
**a** Chemokine and cytokine production in uninfected (medium), *A. phagocytophilum*-infected, IFN-γ-stimulated, *A. phagocytophilum*-infected + INF-γ-stimulated, and LPS-stimulated (200 ng/ml) wild-type Hoxb8 neutrophils at different time points. MIP-1α, RANTES, TNF and IL-6 were measured in the supernatants using CBA assay. Mean and SD from 4 independent experiments are shown. Differences between infected and/or stimulated cells and the medium controls at each time point were analysed using the two-tailed Mann-Whitney test. *P < 0.05. **b** RANTES production of *A. phagocytophilum-*infected, and *A. phagocytophilum*-infected + IFN-γ-stimulated wild-type (WT), IFN-γ R^−/−^ and iNOS^−/−^ Hoxb8 neutrophils. RANTES was measured in the supernatants using CBA assay. Mean and SD from 4 independent experiments are shown. Differences between infected and infected + IFN-γ-stimulated cells at each time point were analysed using the two-tailed Mann-Whitney test. *P < 0.05

The gp91^phox^ and MPO mRNA expression in wild-type cells was unaltered due to IFN-γ stimulation (data not shown). However, the iNOS mRNA expression was significantly induced at time points 24–96 h p.i. (*U* = 0, *n*_1_ = *n*_2_ = 5, *P* = 0.0079 at 24–72 h p.i., Fig. 4d). Compared to unstimulated *A. phagocytophilum*-infected cells a further statistically significant iNOS mRNA increase at time points 24 h, 48 h, 72 h (*U* = 0, *n*_1_ = *n*_2_ = 5, *P* = 0.0079) and 92 h (*U* = 1, *n*_1_ = *n*_2_ = 5, *P* = 0.0159) p.i. was seen when IFN-γ stimulation and infection were combined. However, a significantly elevated nitrite production was detectable only in infected Hoxb8 neutrophils stimulated with IFN-γ at time points 48–96 h p.i. (*U* = 0, *n*_1_ = *n*_2_ = 5, *P* = 0.0079, Fig. 4e). *A. phagocytophilum* infection with or without IFN-γ stimulation led to statistically significant higher amounts of MIP-1α, RANTES, TNF and IL-6 in the supernatants of wild-type Hoxb8 neutrophils compared to the medium controls (*U* = 0, *n*_1_ = *n*_2_ = 4, *P* = 0.0286, Fig. 5a). In infected cells, IFN-γ stimulation led to a significantly higher chemokine and cytokine production. This was most prominent for RANTES at 24–96 h p.i. (*U* = 0, *n*_1_ = *n*_2_ = 4, *P* = 0.0286, Fig. 5b, Additional file 2: Figure S2).

To verify the specificity of the IFN-γ effect, IFN-γ receptor (IFN-γ R) deficient Hoxb8 neutrophils were stimulated with IFN-γ. As expected, the bacterial growth was not affected in IFN-γ R^−/−^ cells due to IFN-γ stimulation (Fig. 4b). A significant induction of iNOS mRNA was not observed in IFN-γ R^−/−^ cells upon IFN-γ stimulation (Fig. 4f). Further, in *A. phagocytophilum-*infected IFN-γ R^−/−^ Hoxb8 neutrophils, there was no significant difference in iNOS mRNA between IFN-γ treated or untreated cells. IFN-γ R^−/−^ Hoxb8 neutrophils showed an unimpaired chemokine and cytokine response upon infection (Additional file 3: Figure S3a) but were unable to produce significant amounts of RANTES and TNF after IFN-γ stimulation (Additional file 3: Figure S3b). Because wild-type Hoxb8 neutrophils did not produce significantly elevated levels of MIP-1α and IL-6 upon IFN-γ stimulation, there were no differences between wild-type and IFN-γ R^−/−^ cells regarding those mediators. An elevated RANTES production in infected cells upon IFN-γ stimulation as seen in wild-type cells was not observed in IFN-γ R^−/−^ Hoxb8 neutrophils when compared to unstimulated infected cells (Fig. 5b). Thus, the observed effects are IFN-γ-specific and need signalling *via* the IFN-γ R.

### Role of iNOS as mediator of the IFN-γ effect

**Fig. 6:**
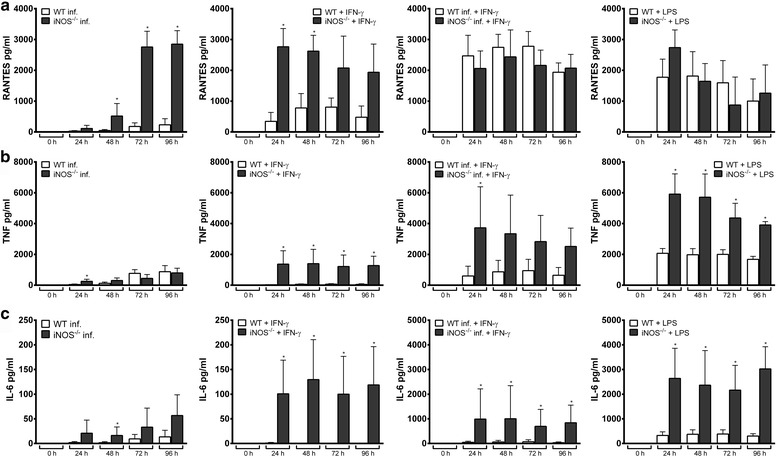
Chemokine and cytokine production of *A. phagocytophilum*-infected, IFN-γ-stimulated, *A. phagocytophilum*-infected + IFN-γ-stimulated, and LPS-stimulated (200 ng/ml) wild-type (WT) and iNOS^−/−^ Hoxb8 neutrophils at different time points. RANTES (**a**), TNF (**b**) and IL-6 (**c**) were measured in the supernatants using CBA assay. Mean and SD from 4 independent experiments are shown. Differences between WT and iNOS^−/−^ cells at each time point were analysed using the two-tailed Mann-Whitney test. *P < 0.05

As we observed that IFN-γ inhibited the growth of *A. phagocytophilum* and simultaneously induced iNOS, we wondered whether the IFN-γ effect was iNOS-mediated. However, the bacterial growth in iNOS^−/−^ Hoxb8 neutrophils was significantly inhibited upon IFN-γ stimulation at 48–96 h p.i. (*U* = 0, *n*_1_ = *n*_2_ = 4, *P* = 0.0286, Fig. 4c). The chemokine and cytokine response in iNOS^−/−^ Hoxb8 neutrophils were generally unimpaired (Additional file 4: Figure S4), but *A. phagocytophilum-*infected iNOS^−/−^ cells produced significantly higher amounts of RANTES than infected wild-type cells (*U* = 0, *n*_1_ = *n*_2_ = 4, *P* = 0.0286, Fig. 6a). The TNF and IL-6 levels were found to be significantly elevated in the supernatants of iNOS^−/−^ Hoxb8 neutrophils upon IFN-γ stimulation when compared to wild-type cells (*U* = 0, *n*_1_ = *n*_2_ = 4, *P* = 0.0286, Fig. 6b, c). In conclusion, this means that the inhibitory effect of IFN-γ on the growth of *A. phagocytophilum* is independent of iNOS and that there might be a compensatory mechanism *via* an increased chemokine and cytokine response.

## Discussion

In the past, in vitro studies on *A. phagocytophilum* have been done primarily using the HL60 cell line or primary human neutrophils [4]. The analysis of primary murine neutrophils is hampered by low yield. Insufficient purity is also an issue because it has been shown that studies on the cytokine production of myeloid cells with purities lower than 98% were unreliable [34]. To overcome these problems, an experimental system has been developed that allows the in vitro generation of murine neutrophils [25]. We used these cells for the first time for the propagation of *A. phagocytophilum*, which grew readily. This supports previous findings that Hoxb8 neutrophils functionally resemble primary murine neutrophils [25–27].

Infected wild-type Hoxb8 neutrophils secreted MCP-1, MIP-1α, RANTES, TNF and after additional IFN-γ stimulation IL-6. These chemokines and cytokines have been shown before to be produced by murine neutrophils in general [30]. Upon *A. phagocytophilum* infection MCP-1, MIP-1α and RANTES were previously found to be secreted by HL60 cells [35]. However, conflicting results were obtained for TNF and IL-6, which were produced by human leukocytes [36], but not by HL60 cells [35]. We did not observe any production of KC, one of the murine IL-8 homologs. In contrast, human neutrophils [37] as well as HL60 cells [35, 37] infected with *A. phagocytophilum* produced IL-8. The differing results concerning the chemokine and cytokine response could have multiple reasons such as usage of murine versus human cells, of primary cells versus cell lines, of various *A. phagocytophilum* strains and of cells of varying purity. However, in conclusion, they show that although *A. phagocytophilum* does not induce the respiratory burst [21–24], it stimulates the chemokine and cytokine response of neutrophils instead of silencing its host cells completely.

The growth of *A. phagocytophilum* was unaltered in gp91^phox−/−^, MPO^−/−^ and iNOS^−/−^ Hoxb8 neutrophils, which is in line with the in vivo finding that gp91^phox−/−^, MPO^−/−^ and iNOS^−/−^ mice were unimpaired in controlling *A. phagocytophilum* [12, 19]. As mentioned above the gp91^phox^, MPO^−^ and iNOS mRNA expression were essentially not changed in the respective gene-deficient Hoxb8 neutrophils infected with *A. phagocytophilum*. Thus, it seems that the effector mechanisms tested here are not significantly involved in compensating for the particular defect. Rather, *A. phagocytophilum* might be insensitive to reactive oxygen or nitrogen species as it has been shown to scavenge O_2_^−^ [23, 24].

Previous reports demonstrated, partially by using non-quantitative PCR techniques that in HL60 cells gp91^phox^ mRNA expression was suppressed upon *A. phagocytophilum* infection [38–40]. However, microarray analyses of infected human neutrophils did not find a downregulation of gp91^phox^ [29, 41]. This is in line with our in vitro and earlier ex vivo results [12].

MPO mRNA was only weakly expressed in uninfected wild-type Hoxb8 neutrophils, probably because MPO synthesis terminates at the myelocyte stage of neutrophil development [31]. Further, MPO mRNA was not induced due to infection at 24–96 h p.i. Others observed MPO mRNA expression in human neutrophils to be downregulated 2-fold at 2 h, but not at 8 h p.i. [42]. In heavily infected sorted human neutrophils, MPO mRNA was suppressed at 24 h p.i. when compared to uninfected neutrophils incubated for 3 h [43]. However, in our hands, incubation alone led to decreased levels of MPO mRNA in uninfected wild-type Hoxb8 neutrophils at 24 and 48 h (Additional file 1: Figure S1d). In HL60 and THP-1 cells, MPO mRNA was found to be downregulated 2.5-fold [44] and 8-fold [45] respectively or remained unchanged at 72 h p.i. [46]. Thus, in conclusion, there seems to be no major alteration of MPO mRNA expression due to *A. phagocytophilum* infection.

In contrast, iNOS mRNA expression was induced 1000-fold in Hoxb8 wild-type neutrophils upon infection. This is contradictory to our ex vivo results, where iNOS mRNA was not differentially regulated in spleen and lung of *A. phagocytophilum*-infected BALB/c mice [12]. The difference might be explained by the low neutrophil content in whole organs. However, in THP-1 cells iNOS mRNA was downregulated 2-fold at 48 h p.i. [45].

IFN-γ is known to induce iNOS [33] and to enhance the bactericidal activity of neutrophils towards extracellular and facultative intracellular bacteria [32]. Elevated IFN-γ levels were found in the sera of humans [18] and mice [11, 12, 15–17, 47] infected with *A. phagocytophilum*. Further, in mice, IFN-γ is important in the early control of *A. phagocytophilum*, although it is dispensable for final elimination [11–14]. We show here that IFN-γ impairs the growth of *A. phagocytophilum* in murine Hoxb8 wild-type cells. Therefore, IFN-γ seems to have a direct effect on an obligate intracellular bacterium that replicates in neutrophils. Several mechanisms how the bacterium partially escapes the IFN-γ dependent immunity have been demonstrated in human neutrophils [48] and in HL60 cells [39] where *A. phagocytophilum* impairs the IFN-γ-induced JAK-STAT signalling and reduces the cell surface expression of CD119 (IFN-γ R α-chain) [48]. Further, in human neutrophils stimulated simultaneously with LPS and IFN-γ *A. phagocytophilum* suppressed the MIG (CXCL9) and IP-10 (CXCL10) production [48]. However, we found that pure IFN-γ stimulation of *A. phagocytophilum*-infected wild-type Hoxb8 neutrophils significantly enhanced iNOS mRNA induction as well as nitrite, RANTES and IL-6 production when compared to unstimulated infected cells. Thus, *A. phagocytophilum* seems not to be able to equally inhibit all IFN-γ-induced pathways.

Although IFN-γ stimulation increased the iNOS mRNA induction in infected cells, the inhibitory effect of IFN-γ on the growth of *A. phagocytophilum* was iNOS independent. Infected and/or IFN-γ-stimulated iNOS^−/−^ Hoxb8 neutrophils produced significantly higher amounts of RANTES, TNF and IL-6 then wild-type cells. It is known that nitric oxide inhibits the expression of cytokines including TNF and IL-6 in myeloid and lymphoid cells [49]. However, in the knock-out situation, it is unclear whether the increased cytokine production compensates in vivo somehow for the defect or whether it just reflects the absence of nitric oxide as negative feedback regulator. Instead of iNOS other IFN-γ regulated effectors such as interferon-inducible GTPases [50] could mediate the growth inhibition of *A. phagocytophilum*. However, in mice, one of them, Irga6, was dispensable in vivo for the control of *A. phagocytophilum* [51]. Thus, other IFN-γ-induced mechanisms have to be investigated in the future.

Irrespective of the underlying mechanism, IFN-γ-stimulated neutrophils seem to contribute to the killing of *A. phagocytophilum*. From our in vivo data, we suggest that in mice the IFN-γ produced in the early phase of infection comes from NK cells [12]. For human and murine neutrophils it has been shown that at least some of their functions can be activated by NK-cell derived INF-γ [52]. Hoxb8 neutrophils infected with *A. phagocytophilum* did not produce IL-12 in vitro. In vivo, we have shown that the control of *A. phagocytophilum* depends on dendritic cells (DCs) [12]. We, therefore, speculate that IL-12 produced by DCs stimulates NK-cells to produce IFN-γ which further activates neutrophils to inhibit the growth of *A. phagocytophilum*. Whether such a DC, neutrophil, NK-cell crosstalk takes place has to be investigated in the future.

## Conclusion

In summary, murine in vitro generated neutrophils stimulated with IFN-γ seem to act not only as host, but as killer cells as well. Although IFN-γ stimulation led to an induction of iNOS, the growth of *A. phagocytophilum* was inhibited by an iNOS-independent mechanism.

## Methods

### Mice

C57BL/6 WT mice were purchased from Charles River Laboratories (Sulzfeld, Germany). C57BL/6 gp91^phox−/−^, C57BL/6 iNOS^−/−^, C57BL/6 MPO^−/−^ and C57BL/6 IFN-γ R1^−/−^ were obtained from the Jackson Laboratories (Bar Harbor, ME, USA). They were housed under specific pathogen-free conditions. The usage of the animals was reported to the Regierungspräsidium Freiburg (X-11/14H).

### Cell lines and cell culture

Female individuals were used at the age of 8 to 12 weeks. Progenitor cells were derived from bone marrow of the mice strains mentioned above. The progenitor cells were retrovirally transduced with estrogen-regulated Hoxb8 and selected for 4 weeks in the presence of stem cell factor (SCF) to generate neutrophil progenitor cell lines [25]. Polyclonal progenitor cell lines were cultured in Opti-MEM + GlutaMAX medium (Life Technologies, Darmstadt, Germany) supplemented with 10% FCS, 30 μM ß-mercapthoethanol, 1 μM ß-estradiol (Sigma-Aldrich, Taufkirchen, Germany) and 1% supernatant from SCF-producing CHO cells. The SCF producing cell line was kindly provided by Hans Häcker (St. Jude Children’s Research Hospital, Memphis, TN, USA). Differentiation was induced by ß-estradiol removal.

### Bacterial strain

The *A. phagocytophilum* Webster strain [53] was routinely grown in differentiated Hoxb8 neutrophils and was passaged every 3 to 4 days. For some experiments, bacteria were cultured in HL60 cells (ATCC CCL-240) in RPMI medium (Life Technologies) with 5% FCS as described [54]. To determine the percentage of infected cells, cells were cytocentrifuged using a Cytospin 4 centrifuge (ThermoFisher Scientific, Langenselbold, Germany) onto glass slides and stained by Diff-Quick (Dade Behring, Marburg, Germany). Two hundred cells were counted at 1000-fold magnification.

### Experimental design

Host-cell free *A. phagocytophilum* obtained from 3 × 10^7^ Hoxb8 neutrophils with an infection rate of 90% was used to infect 1.2 × 10^7^ Hoxb8 neutrophils (differentiated for 4 days) in 6 ml medium. For some experiments, the inoculum was prepared from 3 × 10^6^ HL60 cells with an infection rate of 90%, which was proven to be equivalent to one from 3 × 10^7^ infected Hoxb8 neutrophils. To separate *A. phagocytophilum* from its host cells, the infected Hoxb8 neutrophils were passaged 10 × through a 27 G needle. Subsequently, a differential centrifugation step (10 min 750× *g*, 10 min 2300× *g*) was performed and the pellet used for the infection. Pellets prepared from 3 × 10^7^ uninfected Hoxb8 neutrophils or 3 × 10^6^ uninfected HL60 cells as described above served as control stimuli. At the time points 0 , 24, 48 , 72 and 96 h 500 μl from each set of samples were collected. The pellet was resuspended in RNAlater (Life Technologies) and stored together with the supernatant at -80 °C. Depending on the experiment, cells were stimulated with 10 ng/ml or 200 ng/ml *Escherichia coli* K12 D31m4 (Re) LPS (List Biologicals, Campbell, CA, USA) or 40 ng/ml murine IFN-γ (PeproTech, Rocky Hill, NY, USA). Some set of samples were washed 2× in PBS at 4 h p.i. and were subsequently supplied with fresh medium.

### Quantitative RT-PCR

Total RNA was prepared using TRIzol (Life Technologies), treated with TURBO DNase (Life Technologies) and reverse transcribed with the High Capacity cDNA Reverse Transcription Kit (Life Technologies). Quantitative PCR was performed on an ABI Prism 7900HT Sequence Detector (Life Technologies) using *Taq*Man Gene Expression Master Mix (Life Technologies) and the following assays: gp91^phox^ (Mm00432775_m1), iNOS (Mm00440485_m1), MPO (Mm01298424_m1) and HPRT (Mm00446968_m1). To follow the growth of *A. phagocytophilum* in Hoxb8 neutrophils, the bacterial RNA was quantified using primers 16S RTf2 (5′-GAG AGT TTG ATC CTG GCT CAG AA-3′) and 16S RTr (5′-GCT ATA AAG AAT AAT CCG TTC GAC TTG-3′) and the 16S RT probe (Fam-ACG CTG GCG GCA AGC TTA ACA CAT-BHQ1). Respective mRNA amounts were normalised to murine hypoxanthine guanine phosphoribosyltransferase 1 (HPRT) levels. Relative mRNA expression was calculated using the ΔΔC_t_-method.

### Cytometric bead array (CBA)

Levels of murine IFN-γ, IL-1β, IL-6, IL-10, IL-12/IL-23p40, IL-17A, KC (CXCL1), MCP-1 (CCL2), MIG (CXCL9), MIP-1α (CCL3), RANTES (CCL5) and TNF were measured in the supernatants using CBA Flex Sets (BD Biosciences, Heidelberg, Germany) and a BD LSRFortessa instrument (BD Biosciences). The analysis was performed applying the FCAP array software (BD Biosciences).

### Nitrite accumulation

One hundred microliter supernatant were used to measure nitrite accumulation as an indicator of NO production by Griess reaction with sodium nitrite as standard. The absorbance was measured at 550 nm using an automated plate reader.

### Statistical analysis

Differences between experimental groups were analysed using the two-tailed Mann-Whitney test. Calculations were done by GrapPad Prism 6.05. A *P-*value < 0.05 was considered significant. A correction for multiple testing was not done.

## Additional files

Additional file 1: Figure S1. **a**-**c** Relative gp91^phox^ mRNA expression normalized to murine HPRT at different time points in uninfected, *A. phagocytophilum*-infected and LPS-stimulated (10 ng/ml) wild-type (WT) (**a**), MPO^−/−^ (**b**) and iNOS^−/−^ (**c**) Hoxb8 neutrophils. Results were normalized to the 0 h value of uninfected WT cells using the ΔΔC_t_-method. Mean and SD from 7 independent experiments are shown. Differences between experimental groups were analyzed using the two-tailed Mann-Whitney test. The following groups were compared: infected and LPS-stimulated set of samples to the respective uninfected set of samples at each time point. Statistically significant differences were not detected. **d-f** Relative MPO mRNA expression normalized to murine HPRT at different time points in uninfected, *A. phagocytophilum*-infected and LPS-stimulated (10 ng/ml) WT (**d**), gp91^phox−/−^ (**e**) and iNOS^−/−^ (**f**) Hoxb8 neutrophils. Results were normalized to the 0 h value of uninfected WT cells using the ΔΔC_t_-method. Mean and SD from 7 independent experiments are shown. Differences between experimental groups were analyzed using the two-tailed Mann-Whitney test. The following groups were compared: infected and LPS-stimulated set of samples to the respective uninfected set of samples at each time point. Statistically significant differences were not detected. **g-i** Relative iNOS mRNA expression normalized to murine HPRT at different time points in uninfected, *A. phagocytophilum*-infected and LPS-stimulated (10 ng/ml) WT (**g**), gp91^phox−/−^ (**h**) and MPO^−/−^ (**i**) Hoxb8 neutrophils. Results were normalized to the 0 h value of uninfected WT cells using the ΔΔC_t_-method. Mean and SD from 7 independent experiments are shown. Differences between experimental groups were analyzed using the two-tailed Mann-Whitney test. The following groups were compared: infected and LPS-stimulated set of samples to the respective uninfected set of samples at each time point. *P < 0.05, **P < 0.01, ***P < 0.001. (TIFF 1102 kb)

Additional file 2: Figure S2. Chemokine and cytokine production of *A. phagocytophilum*-infected and *A. phagocytophilum*-infected + IFN-γ-stimulated wild-type (WT), IFN-γ R^−/−^ and iNOS^−/−^ Hoxb8 neutrophils at different time points. MIP-1α (**a**), TNF (**b**) and IL-6 (**c**) were measured in the supernatants using CBA assay. Mean and SD from 4 independent experiments are shown. Differences between infected and infected + IFN-γ-stimulated cells at each time point were analyzed using the two-tailed Mann-Whitney test. *P < 0.05. (TIFF 1038 kb)

Additional file 3: Figure S3. **a** Chemokine and cytokine production in uninfected (medium), *A. phagocytophilum*-infected, IFN-γ-stimulated, *A. phagocytophilum*-infected + INF-γ- stimulated and LPS-stimulated (200 ng/ml) IFN-γ R^−/−^ Hoxb8 neutrophils at different time points. MIP-1α, RANTES, TNF and IL-6 were measured in the supernatants using CBA assay. Mean and SD from 4 independent experiments are shown. Differences between infected and/or stimulated cells and the medium controls at each time point were analyzed using the two-tailed Mann-Whitney test. *P < 0.05. **b** RANTES and TNF production of IFN-γ-stimulated wild-type (WT) and IFN-γ R^−/−^ Hoxb8 neutrophils. RANTES and TNF were measured in the supernatants using CBA assay. Mean and SD from 4 independent experiments are shown. Differences between WT and IFN-γ R^−/−^ cells at each time point were analyzed using the two-tailed Mann-Whitney test. *P < 0.05. (TIFF 758 kb)

Additional file 4: Figure S4. **a** Chemokine and cytokine production in uninfected (medium), *A. phagocytophilum*-infected, IFN-γ-stimulated, *A. phagocytophilum*-infected + INF-γ- stimulated and LPS-stimulated (200 ng/ml) iNOS^−/−^ Hoxb8 neutrophils at different time points. MIP-1α, RANTES, TNF and IL-6 were measured in the supernatants using CBA assay. Mean and SD from 4 independent experiments are shown. Differences between infected and/or stimulated cells and the medium controls at each time point were analyzed using the two-tailed Mann-Whitney test. *P < 0.05. **b** MIP-1α production of *A. phagocytophilum*-infected, IFN-γ-stimulated, *A. phagocytophilum*-infected + IFN-γ- stimulated and LPS-stimulated (200 ng/ml) wild-type (WT) and iNOS^−/−^ Hoxb8 neutrophils. MIP-1α was measured in the supernatants using CBA assay. Mean and SD from 4 independent experiments are shown. Differences between WT and iNOS^−/−^ cells at each time point were analyzed using the two-tailed Mann-Whitney test. *P < 0.05. (TIFF 1068 kb)

## Abbreviations

DC: Dendritic cell
IFN-γ: Interferon-γ
IL: Interleukin
iNOS: Inducible nitric oxide synthase
MPO: Myeloperoxidase
NK: Natural killer cell
p.i.: Post-infection
ROS: Reactive oxygen species
WT: Wild-type
